# A novel assay for monitoring internalization of nanocarrier coupled antibodies

**DOI:** 10.1186/1471-2172-7-24

**Published:** 2006-10-02

**Authors:** Ulrik B Nielsen, Dmitri B Kirpotin, Edward M Pickering, Daryl C Drummond, James D Marks

**Affiliations:** 1 Departments of Anesthesia and Pharmaceutical Chemistry, University of California San Francisco, San Francisco, CA 94110 (UBN, JDM, EMP), USA; 2 Hermes Biosciences, South San Francisco, CA 94080 (DCD, DK), USA; 3 Merrimack Pharmaceuticals, Inc., One Kendall Square, Cambridge, MA 02139, USA

## Abstract

**Background:**

Discovery of tumor-selective antibodies or antibody fragments is a promising approach for delivering therapeutic agents to antigen over-expressing cancers. Therefore it is important to develop methods for the identification of target- and function specific antibodies for effective drug delivery. Here we describe a highly selective and sensitive method for characterizing the internalizing potential of multivalently displayed antibodies or ligands conjugated to liposomes into tumor cells. The assay requires minute amounts of histidine-tagged ligand and relies on the non-covalent coupling of these antibodies to fluorescent liposomes containing a metal ion-chelating lipid. Following incubation of cells with antibody-conjugated liposomes, surface bound liposomes are gently removed and the remaining internalized liposomes are quantitated based on fluorescence in a high throughput manner. We have termed this methodology "Chelated Ligand Internalization Assay", or CLIA.

**Results:**

The specificity of the assay was demonstrated with different antibodies to the ErbB-2 and EGF receptors. Antibody-uptake correlated with receptor expression levels in tumor cell lines with a range of receptor expression. Furthermore, Ni-NTA liposomes containing doxorubicin were used to screen for the ability of antibodies to confer target-specific cytotoxicity. Using an anti-ErbB2 single chain Fv (scFv) (F5) antibody, cytotoxicity could be conferred to ErbB2-overexpressing cells; however, a poly(ethylene glycol)-linked lipid (DSPE-PEG-NTA-Ni) was necessary to allow for efficient loading of the drug and to reduce nonspecific drug leakage during the course of the assay.

**Conclusion:**

The CLIA method we describe here represents a rapid, sensitive and robust assay for the identification and characterization of tumor-specific antibodies capable of high drug-delivery efficiency when conjugated to liposomal nanocarriers.

## Background

Antibodies and antibody fragments can deliver a variety of agents, including drugs, genes, toxins or radioisotopes to target cells expressing the appropriate receptor-antigen. Internalization of the antibody fragment to the interior of the cell can in many cases increase the therapeutic effect of the therapeutic agent [1,2]. A major advantage of receptor mediated internalization as a drug delivery route is that therapeutic agents can be delivered to target cells that specifically overexpress the receptor-antigen and thereby increase efficacy while reducing systemic toxicity. For example, anti-ErbB2 antibodies have been used to target doxorubicin containing liposomes [3,4] or Pseudomonas exotoxin (immunotoxin) into the interior of ErbB2 overexpressing tumor cells [5,6]. A considerable fraction of antibodies generated by immunization do not bind receptors in a manner that triggers internalization [7,8]. Thus, it is desirable to screen for antibodies that can elicit the desired internalization response.

The most common method for monitoring internalization of ligands and antibodies into cells involves radiolabeling of the antibody, incubation of the labeled antibody with the cells, and use of a low pH buffer (usually glycine-HCl pH 2.8) to dissociate surface-bound antibody. However, reports from several laboratories indicate that this buffer in some circumstances only partially dissociates antigen-antibody complexes and therefore can introduce considerable inaccuracies in internalization experiments [9,10]. Alternatively, antibodies can be biotinylated with NHS-SS-biotin and incubated with live cells. Following specific reduction of biotin groups on cell surface bound antibody with reducing agent, the antibody internalization may be quantified by immunoblotting [11]. However, the accuracy of this method also relies on complete removal of biotin from the cell surface bound antibody. In addition, the stringent conditions that are required to strip the cell surface in these procedures may affect cell viability. Another limitation of these methods is that they rely on laborious labeling of each candidate antibody, allowing only a limited number of unique antibodies to be screened for internalization. Finally, the direct labeling of the antibody often results in loss of binding activity to the antigen. These considerable limitations adversely affect both the accuracy and throughput of presently available antibody selection methods and make it desirable to develop a new and more efficient process for screening internalizing antibodies. Here we report about a novel assay for ligand or antibody internalization termed

"Chelated Ligand Internalization Assay" (CLIA), based on a non-covalent attachment of (His)6-tagged ligands to a detectable label bearing a dissociative bond, such as Ni-NTA (nitriloacetic acid) chelation complex. The detectable label consisted of small unilamellar liposomes, thus permitting internalization of multiple reporter molecules in a single internalization event. The liposomes were formulated with Ni-NTA-lipids capable of binding (His)6-tagged proteins. The liposomes bearing Ni-NTA groups on their surface were loaded with fluorescent dye and mixed with a large pool of unique (His)6 containing anti-receptor antibody fragments or intact antibody complexed to (His)6-tagged Protein A.

Internalization of the ligand/liposome/receptor complex was detected by fluorescence microscopy or fluorimetry after gentle removal of cell surface bound complexes using EDTA. Cellular uptake of the complex was dependent on the specificity of the scFv as well as the ability of the antibody fragment to trigger internalization, requiring < 50,000 receptors/cell for detection. The assay required only small amounts (1 μg) of antibody fragment and could be performed using crude, unpurified supernatants of *E coli *that expressed the antibody fragment.

It is also important to translate the observed internalization into cytotoxic readouts. To accomplish this, we used Ni-NTA-conjugated antibody in combination with drug-loaded liposomes to screen for target specific toxicity in breast cancer cells.

Taken together, we describe a new and highly sensitive, selective and robust method for the screening of large arrays of antibodies (e.g. from a phage display library) for specific internalizing ligands that can efficiently deliver agents to selected, receptor-antigen overexpressing cell types.

## Results

### Effect of (His)6-tagged internalizing ligands on the internalization of Ni-NTA liposomes into target cells

**Figure 1:**
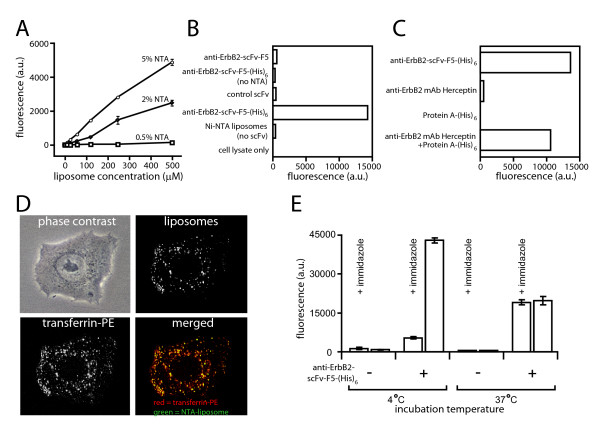
**A**. Effect of liposomal Ni-NTA-lipid concentration on target specific uptake into SKBR3 cells. Liposomes were formulated with entrapped 35 mM HPTS (fluorescent marker) and 0.5, 2 or 5 % Ni-NTA-lipid (% of phospholipid content) and tested for internalization into SKBR3 tumor cells using 20 μg/ml of anti-ErbB2-scFv-F5-(His)6. Liposomes and antibody were not pre-mixed but added sequencially to the media. Liposome concentration, μM liposome phospholipids; fluorescence, relative units. Fluorescence represents a measure of internalization of anti-ErbB2-scFv-F5-(His)6 into SKBR3 cells. **B**. Specificity of the CLIA assay with respect to the presence of (His)6-tagged, internalizing ligand, and fluorescently labeled Ni-NTA-derivatized liposomes.SKBR3 tumor cells were incubated with 500 μM Ni-NTA-liposomes (Ni-NTA = 5% of phospholipid content) and anti-ErbB2-scFv-F5 (without a (His)6-tag), or an irrelevant control scFv antibody (anti-VEGRF2-scFv-4G7-(His)6) or liposomes without scFv (Ni-NTA liposomes (no scFv). Alternatively, anti-ErbB2-scFv-F5-(His)6 was co-incubated with liposomes formulated without the Ni-NTA-DOGS lipid (anti-ErbB2-scFv- F5-(His)6 (no NTA)). All antibody concentrations were 20 μg/mL. The fluorescence was read as a measure of liposome internalization. **C**. Assaying internalization of monoclonal IgG antibodies without (His)6-tag by CLIA using a Protein A-(His)6 chemical conjugate. SKBR3 cells were incubated with anti-ErbB2-scFv-F5-(His)6 (20 μg/mL), with anti-ErbB2 mAb Herceptin (20 μg/mL), Protein A-(His)6 alone (also 20 μg/mL), or mixture of anti-ErbB2 mAb Herceptin and Protein A-(His)6 in the presence of 500 μM fluorescently labeled Ni-NTA-liposomes (Ni-NTA = 5% of phospholipid content). Immunoliposomes were allowed to internalize for four hours. The cells were lysed in base and the fluorescence was read as a measure of internalization. **D**. Co-localization of internalized liposomes with transferrin-PE. Ni-NTA-liposomes, anti-ErbB2-scFv-F5-(His)6 (20 μg/mL), and transferrin-phycoerythrin were co-incubated for two hours with SKBR3 cells before observing cellular localization by fluorescence microscopy using a dual-pass filter. **E**. Temperature dependence of liposome internalization. Ni-NTA liposomes (Ni-NTA = 5% of phospholipid content) were incubated in the presence or absence of ErbB2-scFv-F5-(His)6 (20 μg/mL) at either 4 C or at 37 C for 4 hours. The cells were then washed with either PBS or immidazole and lysed in base and the fluorescence was read as a measure of internalization.

Liposomes were formulated with the entrapped hydrophilic membrane-impermeable fluorescent marker (HPTS) and various amounts of a Ni-NTA lipid (0.5, 2 and 5 % of phospholipid content). They were tested for internalization into ErbB2- overexpressing SKBR3 tumor cells via a highly internalizable anti-ErbB2 scFv antibody (F5) [12] engineered to contain a C-terminal (His)6-tag (anti-ErbB2-scFv-F5-(His)6). Following incubation of SKBR3 cells with the liposomes and the antibody at 37°C, the free and surface-bound liposomes were removed by repeated rinsing with physiological buffer containing Ni-chelating components (1 mM EDTA or 250 mM imidazole). Following a 4h incubation, cells were lysed in basic solution and as a measure of internalization of liposomes the HPTS fluorescence was read in a microfluorimeter. As shown in fig. 1A, the absolute amounts of anti-ErbB2-scFv-F5-(His)6-Ni-NTA-liposome constructs internalized by SKBR3 cells increased with increasing liposome concentration, and with increasing Ni-NTA-lipids in the liposome composition. The cellular uptake of the liposomes was approximately linearly proportional to the concentration of liposomes in the reaction between 0–500 μM phosholipid. Because disintegration or leakage of the entrapped marker would result in substantial reduction or even disappearance of the fluorescent signal, these data also indicate that the Ni-NTA-bearing liposomes were stable enough in the cell culture medium to serve as efficient markers of internalization. Because the dye HPTS is highly charged and highly water soluble it is trapped in the liposome interior. The liposomes are also composed of a lipid composition that does not disintegrate in the presence of plasma (i.e. optimal PC:Cholesterol ratio) and thus the marker would not be expected to be released extracellularly.

The internalization of the liposomes was practically abolished when the added antibody did not contain a (His)6-tag (anti-ErbB2-scFv-F5), when the liposomes were formulated without Ni-NTA-containing lipid (anti-ErbB2-scFv-F5-(His)6 (no NTA)), when the (His)6-tagged antibody was omitted (Ni-NTA liposomes (no scFv)), or when an irrelevant control scFv antibody (anti-VEGFR2-scFv-4G7-(His)6) was used (fig. 1B). Thus, the mixing of an internalizable, (His)6-tagged protein ligand with Ni-NTA-bearing liposomes produced a non-covalent ligand-liposome construct internalizable in a ligand-dependent manner. Moreover, a non-covalent bond between a (His)6-tagged ligand and a Ni-NTA chelating liposome was stable enough to allow the internalization of the construct by ligand-reactive cells under the cell culture conditions.

To expand the utility of the assay to full-length antibodies that do not originally contain a (His)6-tag, we prepared an adapter molecule that could link the Ni-NTA liposomes to the full-length antibodies. Therefore, the antibody binding reagent Protein A was conjugated to the peptide CGGGHHHHHH (C = cysteine, G = glutamine, H = histidine) using the bi-functional reagent sulfo-MBS which cross links the thiol group in the peptide to primary amines on Protein A. SDS-PAGE analysis confirmed successful conjugation of multiple peptides per Protein A molecule as demonstrated by an apparent shift in molecular weight of approximately 10 kDa (results not shown). When SKBR3 cells were co-incubated with Protein A-(His)6 and the monoclonal anti-ErbB2 antibody Herceptin (anti-ErbB2 mAb Herceptin) in the presence of fluorescently labeled Ni-NTA-liposomes, the liposomes were specifically internalized as measured by fluorescence (fig. 1C). Protein A-(His)6 or Herceptin alone did not increase the uptake of Ni-NTA-liposomes, indicating that it is mediated by the Herceptin/Protein A-(His)6 complex (fig. 1C).

The internalized liposomes appear to localize to the endosomes as indicated by the apparent co-localization with fluorescently labeled transferrin and little or no liposomes remain at the cell surface following the EDTA or immidazole wash (fig. 1D). The liposome uptake is temperature dependent and only little uptake is observed when the liposomes are incubated at 4 C (fig. 1E).

### Effect of the antibody concentration: improving the sensitivity of the assay

**Figure 2:**
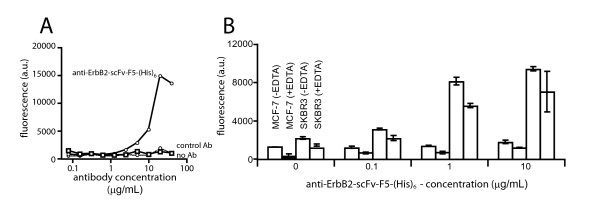
**A**. Effect of the Ni-NTA liposome concentration in the CLIA assay. SKBR3 cells were co-incubated with (squares) or without (circles) 20 μg/mL of the anti-ErbB2-scFv-F5-(His)6 and varying concentrations of fluorescently labeled Ni-NTA-liposomes (Ni-NTA = 2% of phospholipid content). Internalization of liposomes was measured in a microfluorimeter. **B**. Effect of antibody concentration in the CLIA assay. SKBR3 cells were coincubated with 500 μM of fluorescently labeled Ni-NTA-liposomes containing 2 % of phospholipid content and varying concentrations of either the anti-ErbB2-scFv-F5-(His)6 (solid line-circles), or an irrelevant control antibody (anti-VEGRF2-scFv-4G7-(His)6)(squares), or with no antibody (dotted line – circles). **C**. Effect of the anti-ErbB2-scFv-F5-(His)6 concentration on the uptake of fluorescently labeled, minimally PEGylated Ni-NTA-liposomes (Ni-NTA = 5% of phospholipid content; 0.5 mol.% PEG(M.w. 2,000)-DSPE) by cells with high (SKBR-3 cells) or low (MCF-7 cells) expression of ErbB2 receptor. (-EDTA), cells were washed with Hanks' BSS without EDTA; (+EDTA), cells were washed with Hanks' BSS + 1 mM EDTA. Experiments were done in duplicate. Error bars, SD. For all the experiments, immunoliposomes were allowed to internalize for four hours. The cells were lysed in base before reading the fluorescence in a microfluorimeter. Incubation time was 4 hours.

The sensitivity of the assay was tested by incubating SKBR3 cells (naturally overexpressing ErbB2) with 500 μM of the HPTS-loaded liposomes containing 2% Ni-NTA (of phospholipid content) and varying concentrations of anti-ErbB2-scFv-F5-(His)6 (fig. 2A). Only increasing concentrations of the antibody resulted in the gradual increase of the liposome internalization of the complex. As expected, when the irrelevant anti-VEGFR2- scFv-4G7-(His)6 control antibody (VEGFR2 is not expressed in significant amounts on SKBR3 cells) was substituted for anti-ErbB2-scFv-F5-(His)6 it did not mediate internalization of the NTA-liposomes above the blank samples containing no antibody (fig. 2A). The detection limit of the assay with the anti-ErbB2-scFv-F5-(His)6 on SKBR3 cells was about 7 ug/ml.

In an attempt to increase the sensitivity of the assay, we formulated Ni-NTA-liposomes with the following lipid matrix composition: POPC, 25 molar parts; DOGS-NTA-Ni, 5 molar parts; cholesterol, 20 molar parts; and PEG(2000)-DSPE, 0.5 molar parts. Higher concentration of DOGS-NTA-Ni (Ni-NTA = 20% of phospholipid content) was expected to increase the sensitivity, while inclusion of a PEGylated lipid PEG-DSPE at low amount (1 mol.% of the total lipid) was to reduce liposome aggregation and potentially increase the non-specific cell-liposome interactions due to increased Ni-NTA-lipid content. These liposomes were similarly tested on ErbB2-overexpressing SKBR3 cells (about 10^6 ^receptors/cell) and on MCF-7 cells with low ErbB2 expression (about 33-fold less than SKBR3 cells; fig. 2B).

Following 4 hour incubation with the liposomes (0.5 mM phospholipid) in the cell growth media in the presence of 0.1–10 μg/mL of anti-ErbB2-scFv-F5-(His)6, the cells were washed 4 times with Mg2+- and Ca2+-containing Hanks' BSS (to prevent cell detachment during washes). The amounts of cell-associated liposomes were compared in the samples after receiving two additional washes, either with Mg2+- and Ca2+-free Hanks' BSS ("-EDTA"), or with PBS containing 1 mM EDTA ("+EDTA"). The HPTS fluorescence in alkaline lysates of the cells washed without EDTA reflected the totality of cell-internalized and surface-bound liposomes, while the fluorescence of the samples obtained after the EDTA wash was assumed to be proportional to the internalized liposome fraction (fig. 2B).

In MCF-7 cells, the increasing amounts of anti-ErbB2-scFv-F5-(His)6 to 10 μg/mL resulted in an approximately 20% increase in the amount of cell-associated liposome marker over the background (no antibody) control, which was not statistically significant (*p*>011 by Student's t-test). In SKBR3 cells, the amount of cell-associated liposomes was still not significant at 0.1 μg/mL of anti-ErbB2-scFv-F5-(His)6, but at both 1 μg/mL and 10 μg/mL of anti-ErbB2-scFv-F5-(His)6, there was a statistically significant (*p *< 0.03) increase of the liposome uptake by SKBR3 cells to 150–200% over the background. The fluorescence ratio between "+EDTA" and "-EDTA" samples indicated that 73–79% of the cell-associated liposome-antibody constructs were inaccessible to EDTA washing, i.e., internalized. This value was in good agreement with the previously determined degree of anti-ErbB2-scFv-F5-(His)6 internalization into SKBR3 cells (about 80%) using the traditional method of 125I-labeled antibody and acid wash [13]. Thus, the improved liposome composition allowed an increase in the assay sensitivity to the equivalent of about 1 μg/mL of a single chain Fv antibody.

### CLIA does not require antibody purification

**Figure 3:**
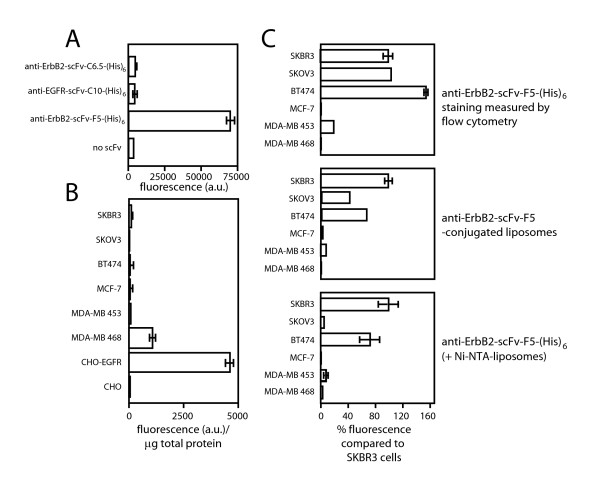
**A**. Internalization of unpurified, bacterially produced antibodies into SKBR3 cells. SKBR3 cells were co-incubated with supernatants of *E. coli *expressing anti-EGFR-scFv-C10-(His)6, the non-internalizing anti-ErbB2-scFv-C10-(His)6, anti-ErbB2-scFv-F5-(His)6, or no scFv along with fluorescently labeled Ni-NTAliposomes (500 μM, Ni-NTA = 2% of phospholipid content). The fluorescence represents the amount of internalized antibody. **B**. Tumor cell profiling of EGFR with anti-EGFR-scFv-C10-(His)6 and Ni-NTAliposomes. Anti-EGFR-scFv-C10-(His)6 was co-incubated with fluorescently labeled Ni-NTA-liposomes and cell lines expressing varying amounts of EGFR: SKBR3, SKOV3, BT474, MCF7, MD-MBA 453, MD-MDA 468, CHO-EGFR, or CHO. The fluorescence represents the uptake of labeled liposomes. The uptake of fluorescently labeled liposomes was normalized to total cellular protein. **C**. Comparison of ErbB2 expression levels and uptake of anti-EGFR-scFv-F5-(His)6-conjugated immunoliposomes or anti-EGFR-scFv-F5-(His)6 chelated liposomes (anti-EGFR-scFv-F5-(His)6(+Ni-NTA-liposomes)). ErbB2 expression levels on cells were determined using fluorescently labeled anti-EGFR-scFv-F5-(His)6 and flow cytometry (top panel). Alternatively, live tumor cells were incubated with anti-ErbB2-scFv-F5-(His)6 covalently coupled to fluorescently labeled liposomes (anti-ErbB2-scFv-F5-(His)6-conjugated liposomes) (middle panel). The CLIA assay was performed by co-incubation of anti-ErbB2-scFv-F5-(His)6 and fluorescently labeled Ni-NTA liposomes (bottom panel). Liposome fluorescence was read in a microfluorimeter. The fluorescence scale in all 3 panels is indicated as % of SKBR3 signal which was set to 100 %.

Because of the specific interaction of the (His)6-tag with Ni-NTA on the liposome, we hypothesized that the assay would permit the use of unpurified scFv recombinant antibodies, even crude preparations such as bacterial lysates, hybridoma media or ascites fluid, allowing a large number of scFv antibody variants to be screened for internalization. To test this hypothesis, soluble scFv expression was induced from *E. coli *in 96-well culture plates and the supernatant tested for activity on live SKBR3 cells using 5% fluorescently labeled Ni-NTA liposomes. In previous experiments (results not shown) we have determined that SKBR3 cells tolerate as much as 50 % bacterial culture supernatant for up to 24 h. Bacterial supernatants from culture plates of *E coli *expressing the anti-ErbB2-scFv-F5-(His)6 were mixed 1:3 (vol:vol) with cell culture media containing 10 % serum, antibiotics, and 500 μM HPTS-labeled Ni-NTA-liposomes (Ni-NTA = 5% of phospholipid content). The mixture was subsequently incubated with live SKBR3 cells. The amount of cell-internalized liposomes -measured by fluorescence- was similar to results obtained with 20 μg/mL of purified anti-ErbB2-scFv-F5-(His)6 and exhibited similar specificity (results not shown). Interestingly, the non-internalizing anti-ErbB2-scFv-C6.5-(His)6 did not result in uptake of NTA liposomes. This is consistent with previous results obtained by confocal microscopy analysis of the internalization of anti-ErbB2-scFv-F5-(His)6 and anti-ErbB2-scFv-C6.5-(His)6 [37]. For some antigens (e.g. some integrins), multimerization is known to induce internalization; however, this data suggests that this is not the case with NTA lipomes bound to ErbB2. A (His)6-tagged EGFR-specific internalizable single chain Fv C10 (anti-EGFR-scFv-C10-(His)6) [38] used as a control did not cause a noticeable internalization of the liposomes over the blank sample (antibody-free microbial supernatant, no scFv), consistent with the low amount of EGF receptors expressed by SKBR3 cells (fig. 3A).

### Profiling tumor cell lines for antibody internalization

In addition to screening antibodies for internalization by a given cell line, the assay allowed screening of cell lines for their capacity to internalize a given antibody. As an example, anti-EGFR-scFv-C10-(His)6 was used to profile a panel of breast cancer cell lines and CHO transfectants (fig. 3B). Only the cell line MD-MDA 468 and CHO cells transfected with EGFR internalized significant amounts of NTA-liposomes. The specificity of the assay is exemplified by anti-EGFR-scFv-C10-(His)6 internalizing into CHO cells transfected with EGFR, but not untransfected CHO cells. Uptake of the fluorescent NTA liposomes into the EGFR transfected CHO cells was 165 times that of untransfected cells (fig. 3B).

The profile of anti-ErbB2-scFv-F5-(His)6 internalization largely correlated with cell surface expression of ErbB2 as determined by flow cytometry with anti-ErbB2-scFv-F5-(His)6 (fig. 3C). However, the cell line SKOV3 did not take up as many liposomes as would be expected from its cell surface expression level of ErbB2. The poor internalization of ErbB2 into this cell line has been described previously [4]. When total uptake into the same panel of cell lines was determined using anti-ErbB2-scFv-F5-liposomes (in which the antibody is covalently coupled to the lipid) the discrepancy with anti-ErbB2-scFv-F5-(His)6 binding determined by flow cytometry was less pronounced. Anti-ErbB2-scFv-F5-(His)6 linked covalently to the liposome surface is not removed by the EDTA washes used to disrupt the chelation of (His)6-tagged scFv to Ni-NTA chelating liposomes. This is most likely due to cell surface bound anti-ErbB2-scFv-F5-(His)6-liposomes. These results suggest that the dissociating bond between the detectable marker (a liposome) and the antibody is required for an effective measurement of internalization.

### Cytotoxicity of targeted liposomal therapeutics

Another potential application of the CLIA assay is the screening and characterization of target-specific cytotoxic activity of immunotargeted liposomal therapeutics. Since different internalization pathways may lead to differences in cytotoxicity of the immunoliposome the ability to rapidly test combinations of targeting molecules and drug-loaded liposomes is of interest.

**Figure 4:**
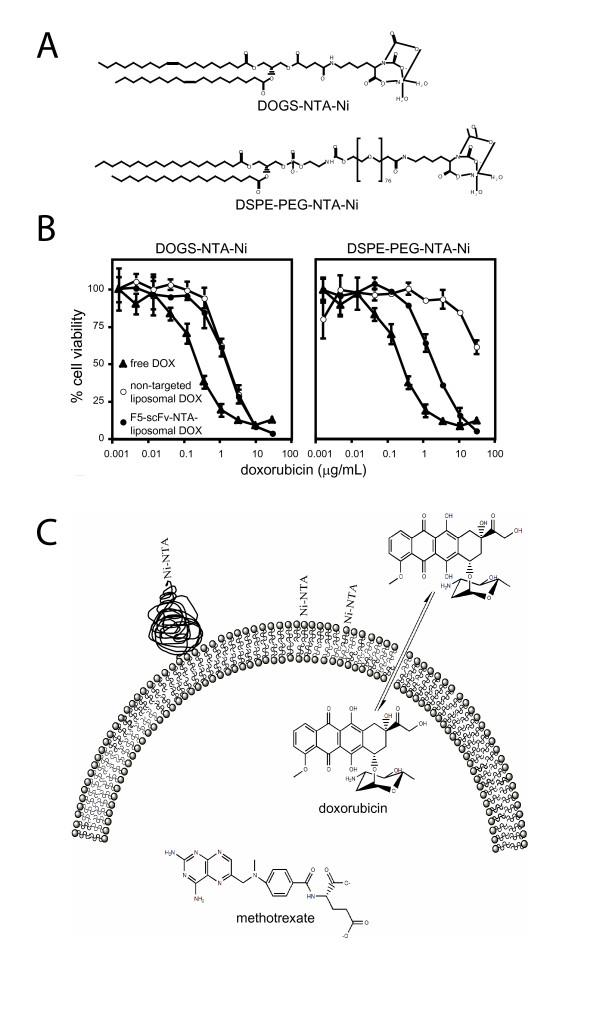
**A**. Chemical structures of DOGS-NTA-Ni and DSPE-PEG-NTA-Ni. DOGS-NTANi is an acidic lipid derivative with the functional moiety located immediately at the membrane surface where it can potentially interact with amphipathic drugs, such as doxorubicin, that may be situated in or be transversing the membrane. DSPE-PEG-NTA-Ni, on the other hand, has the NTA-Ni separated from the membrane by a poly(ethylene glycol) (PEG) spacer where interactions with drugs will likely be minimized. Doxorubicin (DOX) is commonly carried in the liposomal lumen in a precipitated form. However, it must transverse the membrane in order to be actively loaded into the liposomes. Highly water soluble drugs, including methotrexate (MTX), are encapsulated passively and reside almost exclusively in the liposomal interior. **B**. Doxorubicin-dependent cytotoxicity of anti-ErbB2-scFv-F5-(His)6 coupled to different liposomes. In each of these graphs, free doxorubicin (▲) is compared to anti-ErbB2-scFv-F5-(His)6-NTA-liposomal-DOX (●) and non-targeted NTAliposomal DOX with or without PEG spacer (○). Cytotoxicity was determined using a calcein AM-cell viability assay as described in the methods section.

To test this application, we prepared fluorescently labeled liposomes containing DOGS-NTA-Ni (Ni-NTA = 2% of phospholipid content) and loaded them with the cytotoxic drugs vinorelbine, methotrexate, or doxorubicin. Methotrexate was loaded passively and achieved a loading efficiency of approximately 30 %, equivalent to liposomes containing no DOGS-NTA-Ni (table 1). Doxorubicin and vinorelbine were loaded at the drug/phospholipid ratio of 150 mg/mmol using an ammonium sulfate gradient-based remote-loading method. This method typically results in loading efficiencies of 95–100 % for these drugs with neutral phospholipid liposomes. However, in the presence of DOGS-NTA-Ni, the loading efficiency was reduced to about 26.9% for doxorubicin and 9 % for vinorelbine. We hypothesized that the reduced loading resulted from an interaction of the amphipathic drugs with the NTA-Ni functionality located at the membrane surface. Unexpectedly, the high loading efficiency (92–100%) was restored when a different NTA-Ni-lipid derivative, DSPE-PEG-NTA-Ni (NTA-Ni linked to the lipid anchor via a PEG spacer), was substituted for DOGS-NTA-Ni (fig. 4A). Liposomes with DSPE-PEG-NTA-Ni instead of DOGS-NTA-Ni reached a loading efficiency of 92–100 %, similar to that seen with nonchelating liposomes. These results suggest that extending the NTA-Ni chelating group away from the membrane surface will be important for forming liposomes where the drug is loaded via remote-loading methodology, or where the drug either transiently or permanently resides in the membrane.

**Table 1:** Effect of Ni-NTA chelating lipids on the efficiency of loading for various anticancer drugs into liposomes.

Chelating lipid	Drug loaded^1^	Drug-to-lipid^2 ^ratio – input (μg/μmol)	Drug-to-lipid^3 ^ratio – final (μg/μmol)	Loading^4 ^efficiency (%)
none	DOX	150	159.4 ± 6.1	106.3 ± 14.4
Ni-NTA-DOGS	DOX	150	40.4 ± 4.3	26.9 ± 3.0
Ni-NTA-PEG-DSPE	DOX	150	138.2 ± 10.2	92.2 ± 8.0
				
none	VRB	150	150.2 ± 4.9	100.2 ± 3.4
Ni-NTA-DOGS	VRB	150	13.6 ± 1.2	9.0 ± 0.9
Ni-NTA-PEG-DSPE	VRB	150	149.6 ± 5.8	99.8 ± 4.0
				
none	MTX	508	146.0 ± 20.3	28.7 ± 4.0
Ni-NTA-DOGS	MTX	508	149.8 ± 6.6	29.5 ± 1.3
Ni-NTA-PEG-DSPE	MTX	508	151.1 ± 7.2	29.7 ± 2.0
				

^1^Doxorubicin (DOX), vinorelbine (VRB), and methotrexate (MTX) were quantitated by absorbance at 498, 270, and 350 nm, respectively, following dissolution of the liposome sample in acid isopropanol.

^2^Phospholipid was determined by phosphate analysis as described by Bartlett, 1959

^3^Free drug was removed by Sephadex G-75 gel filtration chromatography.

Cytotoxicity studies with liposomal doxorubicin demonstrate target specific activity for liposomes in the presence of 20 μg/mL anti-ErbB2-scFv-F5-(His)6 (scFv-F5-NTA liposomal DOX, fig. 4B). The cytotoxicity of DSPE-PEG-NTA-Ni-liposomes containing doxorubicin (non-targeted liposomal DOX, fig 4B, right panel) increased 26-fold in SKBR3 cells, but not of liposomes prepared with DOGS-NTA-Ni (non-targeted liposomal DOX, fig. 4B, left panel). The difference in the IC50 between targeted and nontargeted liposomal doxorubicin in the PEG-linked construct was 26-fold, 1.92 vs. 49.6 μg/mL, respectively. The IC50 for the free drug (free DOX) was 0.25 μg/ml (fig. 4B, right panel). The cytotoxicity of doxorubicin-loaded DOGS-Ni-NTA-containing liposomes did not change in the presence of anti-ErbB2-scFv-F5-(His)6 (IC50 = 1.73 μg/mL in both cases, fig. 4B, left panel) which likely reflects the higher drug leakage rate from these liposomes, consistent with their decreased drug-loading efficiency. Thus, accurate screening of antibodies for target-specific activity using liposomal drug constructs significantly benefited from using a PEGylated lipid that may act as a spacer to move the chelating group away from the membrane surface, at least when amphipathic drugs such as doxorubicin are employed. The PEG spacer also provides the potential for insertion of the chelating lipid into the outer monolayer of drug-loaded or fluorescentlylabeled liposomes, thus transforming a nontargeted liposome into a targeted one upon addition of (His)6-containing scFvs.

## Discussion

Antigen-specific antibody fragments can be directly selected using phage display libraries where the fragments are displayed on the surface of filamentous bacteriophages [14]. These libraries can generate panels of unique scFv antibodies with varying binding affinities to virtually any antigen [15]. The selected antibody fragments can be used for a wide variety of therapeutic applications, including directly altering normal antigen function [16,17] and targeted delivery of radioisotopes, toxins [20,21], small molecule drugs, liposomal drugs [2], and direct effector cells to tumor sites [19]. The activity of many of these immunotargeted therapies, including immunotargeted nanocarriers for nucleic acids [12], immunoliposomes [2], and immunotoxins [20], often rely on intracellular delivery for optimal activity. Internalization of the immunoliposomal drugs is thought to result in intracellular release of the drug from the carrier, a more uniform intratumoral distribution, and decreased diffusion of the drug from the therapeutic targets [24,27,28]. Thus, the preferential selection and characterization of internalizing ligands would aid in the development of certain therapeutics that depend on internalization for "activation" and thus optimal in vivo efficacy.

A wide variety of methods have been described for selecting antigen-specific antibodies from phage display libraries, most often related directly to antigen binding [14,29-35]. Selection can be to antigen coated on plates, on column matrices, cells displaying the antigen, or to antigen in solution followed by capture onto a solid support. Alternatively, phage display libraries can be screened for antibodies that elicit a particular response, such as internalization [36-38]. Using this method, a series of unique and internalizing anti-ErbB2 [36,37] and anti-EGFR [38] scFv antibodies have been identified and are currently being utilized in our laboratories for the development of targeted therapeutics [2,39] but the method is limited in scope because it does not allow quantitation of internalization nor does it characterize antibody internalization into multiple cell lines.

Due to the increasing need for identification of internalizing ligands for therapeutic or diagnostic applications, the development of sensitive and accurate methods for screening or characterizing antibodies for internalizing capabilities is essential. Current methods are filled with inaccuracies, often due to incomplete removal of surface bound ligand [9-11]. Other limitations involve loss of binding activity upon ligand labeling, inefficient labeling, or poor sensitivity.

Here we have described the development of a novel assay that uses histidine-tagged antibody fragments bound to Ni-NTA-lipid anchored liposomes encapsulating a membrane impermeable fluorophore to monitor cellular internalization. We call this method "Chelated Ligand Internalization Assay", or CLIA. The use of Ni^2+^-chelates to bind (His)6-tagged proteins has been commonly employed in the purification of recombinant proteins [40]. Indeed, we often use this methodology for purification of selected scFvs, to be used in different targeted therapeutic and diagnostic applications. Chelating lipids, most commonly lipid-modified derivatives of nitrioltriacetic acid, have been used to study receptor-ligand interactions in model membranes [41], for two-dimensional crystallization of histidinetagged proteins [42], for capturing modified peptides on membranes for western blot analysis [43] or for reversible binding of proteins to membranes [44-46]. They have also been used in early studies of potential therapeutic approaches, including attaching costimulatory molecules to membrane vesicles for tumor immunotherapy [47,48] or modification of liposomes with various ligands such as an anti-HER2 peptide [49].

Because internalization can be significantly affected by the size and valency of ligand display for the internalizing conjugate, the method described here can not be generalized to conjugates of widely variable sizes and/or degrees of ligand display. The multivalent display of receptor ligands or antibodies specific for cell-surface receptors can dramatically increase avidity for its receptor compared to the corresponding monovalent ligand [57], result in cross-linking of cell surface receptors [73,59], and have an effect on the efficiency of internalization into cells [23,72]. Indeed we have observed an increased internalization efficiency when scFv are displayed multivalently on the surface of phage [23]. However, conjugate binding and internalization may also reach a plateau at a certain valency and then decrease in efficiency at higher valencies [68,2]. Finally, multivalent display of ligands can affect the intracellular trafficking of the ligand or ligand-conjugate [63,58,71].

The size of the conjugate may also play a role in determining the efficiency of internalization. The optimum diameter for spherical particles with respect to internalization appears to be in the range of 50–60 nm [64,66,60]. However, particles up to 500 nm can be readily taken up into nonphagocytic eukaryotic cells, with increasing size from 50–200 nm giving rise to a reduced efficiency of internalization, and particles of higher size being taken up by a distinct mechanism (i.e. caveolae) [74]. Ligand-targeted liposomes are typically prepared in the optimum range of 80–120 nm due to the reduced clearance of small relative to large particles in vivo, and the inherent instabilities observed in drug encapsulation at very small sizes where a high radius of curvature can result in increased membrane permeability to encapsulated agents [65,67].

In this manuscript we describe a method for characterizing and screening multivalently displayed ligands using scFv fragments conjugated noncovalently to the surface of liposomes through a hexahistidine tag in the C-terminus of the scFv. The liposomes have been designed to minimize leakage or disassociation of the fluorescence probe from the carrier. HPTS is a highly charged and water soluble aqueous contents marker that is stably retained in the liposomal interior [62] and has been widely utilized in the liposome field to characterize the interactions of liposomes with cells, specifically with respect to internalization [70,62,4]. Great care must be chosen in the choice of probe. Some lipid-soluble fluorescent dyes are readily extractable by plasma proteins or cellular membranes while some fluorescent aqueous contents markers (carboxyfluorescein) can rapidly leak from the liposome at the low pH experienced during internalization due to protonation of the flurophores carboxyl groups [69,61]. Indeed, the later studies with encapsulated cytotoxic agents illustrate this point, as doxorubicin encapsulated in a less stable formulation gives rise to nonspecific cytotoxicity (Figure 4).

The final studies in this manuscript describe a modification of the initial CLIA method for characterizing the cytotoxicity of ligand-targeted immunoliposomal therapeutics. Because the main purpose of the methodology is to identify suitable therapeutic targeting molecules, it is necessary to screen various targeting ligands for their ability to elicit the desired effect. Although internalization is generally known to be required for a substantial improvement in antitumor activity, it is important to understand that different targeting ligands and different receptors may traffic to very different intracellular pathways thus resulting in differences in cytotoxic activity for the delivered drug that depend on the targeting ligand employed. We have recently observed in the same cell line, cytotoxic activity that varied from 20–50 fold between immunoliposomes prepared using two different scFv targeting ligands, despite the fact that they resulted in similar levels of internalized liposomes (data not shown). Although it is possible to proceed directly to screening for cytotoxicity, this screening is considerably more cumbersome due to the fact that a series of dilutions is required to accurately determine the MTD for the drug. It is thus more desirable to reduce the number of scFv antibodies to be screened for targeted cytotoxicity based on the results of the more high throughput CLIA assay. However, in order to accurately screen for differences in cytotoxic activity it was necessary to modify the liposome construct to include a Ni^2+ ^chelate that was sufficiently removed from the surface of the carrier so as not to interfere with drug encapsulation, or subsequent stability during the course of the assay.

## Conclusion

To our knowledge, this is the first study to use this coupling methodology as a tool to quantitate receptor-mediated internalization by taking advantage of the reversible nature of the noncovalent Ni-(His)6 linkage. We have found that (His)6-tagged scFvs bind to liposomes containing these modified lipids and give sensitive reporting of receptor-mediated internalization and target-specific cytotoxic activity. In this work, we have also described the synthesis of a new Ni-NTA-lipid chelate, DSPE-PEG-NTA-Ni, containing a poly(ethylene glycol) linker for preparing stable liposomal drug constructs with the capability of rapidly binding his-tagged proteins. The low amount of antibody required and the robust nature of the assay allow for high throughput screening of internalizing ligands. In addition, the application of this assay to cytotoxicity assessments allows us to directly screen antibodies for the target-specific activity that is often the ultimate goal of an application for these molecules. We believe this technology will prove invaluable for the rapid discovery and development of targeting ligands against novel therapeutic targets.

## Methods

### Materials

1-palmitoyl-2-oleoyl-phosphatidylcholine(POPC), distearoylphosphatidylcholine (DSPC), N- [methoxy(polyethyleneglycol)-2000]-1,2-distearoyl-phosphatidylethanolamine (PEG-DSPE), and 1,2-dioleoyl-sn-glycero-3- [N(5-amino-1-carboxypentyl) iminodiacetic aicd]succinyl] (nickel salt) (DOGS-NTA-Ni) were purchased from Avanti Polar Lipids (Alabaster, AL). Cholesterol was obtained from Calbiochem (San Diego, CA); organic solvents, HPLC grade were from Fisher; Pittsburgh, PA; and other chemicals of reagent purity from Sigma Chemicals; St. Louis, MO. Vinorelbine (GlaxoSmithKline; Triangle Park, NC), and doxorubicin (Bedford Laboratories; Bedford, OH) were obtained commercially from the pharmacy. Methotrexate was purchased from Sigma Chemicals (St. Louis, MO). Pyranine, or 8-hydroxypyrene-1,3,6-trisulfonic acid (HPTS), and calcein-acetyoxymethyl ester (calcein-AM) were purchased from Molecular Probes (Eugene, OR). Anti-VEGFR-scFv-4G7-(His)6 was a kind gift from Udo Geissler. Anti-ErbB2 mAb Herceptin was obtained form the pharmacy.

### Liposome preparation

Liposomes were prepared from POPC and cholesterol (3:2 ratio), with varying amounts of chelating lipids DOGS-NTA-Ni or DSPE-PEG-NTA-Ni (see below) and in some cases also with a PEG lipid PEG-DSPE. (Avanti Lipids; 0.5–5% of phospholipid content). The lipid components were co-dissolved in chloroform, and the solution was evaporated under reduced pressure to form a lipid film. The lipid film was hydrated in an aqueous solution containing 0–35 mM of the fluorescent marker HPTS (Molecular Probes), adjusted to pH 7.0 with NaOH and the osmolality of about 280 mOs mM/kg with NaCl. Following hydration, the liposomes were formed by membrane extrusion 10–12 times through two stacked 0.1 μm polycarbonate membranes (Corning) as described [4], and unencapsulated HPTS was removed by gel-filtration on a Sephadex 25 G-75 column using HEPES-buffered saline (HBS; 20 mM HEPES, 144 mM NaCl. pH 7.2).

For encapsulation of the chemotherapy drugs doxorubicin and vinorelbine, liposomes were formed in the presence of 250 mM ammonium sulfate and loaded using an ammonium sulfate remote loading method [50,51]. Briefly, following extrusion of the lipid suspensions through two stacked 0.1 μm polycarbonate filters, unencapsulated ammonium sulfate was removed on a Sephadex G-75 column eluted with Mes-buffered saline (pH 5.5). The phospholipid content was then determined by phosphate analysis [52] and either vinorelbine or doxorubin was added at a ratio of 150 μg drug/μmol phospholipid. The drug was loaded by incubation with the liposomes at 58°C for 45 min and subsequent quenching of the reaction on ice for 15 min. Unencapsulated drug was removed on a second Sephadex G-75 gel filtration column. Loading efficiencies were typically in the range of 95–100 % when 150 μg drug per μmol phospholipid was used. Methotrexate-loaded liposomes were prepared by passive encapsulation in an aqueous solution containing methotrexate (200 mM, 5 mM HEPES, pH 7.0). The amount of drug in the purified liposome samples was quantitated by absorbance (doxorubicin; OD498; vinorelbine, OD270; methotrexate, OD298) following dissolution of the drug-loaded liposomes in acid isopropanol (90 % isopropanol, 10 % 1 N HCl), and phospholipid content was determined by standard phosphate analysis [52]. Drug loading efficiencies were calculated both in absolute amounts where they are expressed as μg of drug/μmol of phospholipids and in relative terms, where they are expressed as the % of drug loaded relative to the initial amount of drug added. The average liposome size was determined by photon correlation spectroscopy, and was typically in the range of 105–120 nm for liposomes prepared under these conditions.

### ScFv expression and purification

The scFv's C6.5 (anti-ErbB2) [53], and F5 (anti-ErbB2) [37] were cloned into expression vector pUC119mycHis [53] and expressed in *E. coli *TG1. Briefly, 0.75 L of media (2xTY with 100 μg/mL ampicillin and 0.1% glucose) were inoculated 1/100 with an overnight culture. The culture was grown to an A600 of 0.9 and expression was induced by the addition of isopropyl-b-D-thiogalactopyranoside (IPTG) to a final concentration of 0.5 mM. The culture was then incubated at 30°C for an additional four hours.

Cells were harvested by centrifugation (4000 × g, 20 min.) and the pellets were resuspended in periplasmic extraction buffer (PPB) (30 mM Tris, 2 mM EDTA, 20% sucrose, pH 8.0) containing DNase (100 μg/mL) and incubated on ice for 30 min. The suspension was spun at 5000 × g for 20 min. The pellets were resuspended in osmotic shock buffer (5 mM MgSO4) and incubated for another 20 min on ice. The suspension was spun (7000 × g, 20 min.) and supernatants from the PBB and MgSO4 fractions were combined and cleared by centrifugation at 10000 rpm for 30 min at 4°C. The resulting solution was dialyzed in PBS (two changes, 4 L PBS, pH 8.0). All antibodies were purified by immobilized metal affinity chromatography (IMAC) (Qiagen) followed by desalting on a PD10 column (Pharmacia). Protein concentrations were determined spectrophotometrically (absorbance at A280) using the extinction coefficient ε = 1.4. For induction in microtitre plates, wells containing 150 μl of 2 × TY containing 100 μg/ml ampicillin and 0.1% glucose were inoculated with an overnight culture of TG1 with the plasmid containing the scFv. Cultures were grown to an A600 ~ 1, and scFv expression induced by the addition of IPTG to a final concentration of 1 mM. Bacteria were grown overnight at 30°C, centrifuged, and 30 μL of the supernatant containing scFv used directly in the internalization assay.

### Preparation of protein A-(His)6 conjugate

Protein A was conjugated to the (His)6-containing peptide CGGGHHHHHH using the bifunctional reagent *m*-maleimidobenzoyl-*N*-hydroxysulfosuccinimide ester (Sulfo-MBS; Pierce). Protein A (2 mg) was treated with Sulfo-MBS (0.2 mg) in PBS for one hour at room temperature. Free Sulfo-MBS was removed by gel filtration chromatography and the protein then reacted with 0.2 mg of the (His)6-containing peptide in phosphate buffered saline (PBS) for one hour at room temperature. Free peptide was removed by gel filtration chromatography.

### CLIA assay procedure

Human breast cancer cells SKBR3, SKOV3, BT474, MCF7, MDA-MB-453, MDA-MB-468 (American Type Culture Collection) were grown to 80–90% confluence in the media type recommended by ATCC supplemented with 10% fetal calf serum (FCS) and harvested by trypsinization using standard techniques. Cells (10,000) were seeded in 96-well plates and incubated overnight at 37°C. The next day, Ni-NTA liposomes (0 – 1 mM total phospholipid) were incubated for 4 h with the cells along with the (His)6-containing ligand (20 μg/mL unless otherwise indicated) in 100 μL tissue culture media supplemented with 10 % FCS. Liposomes and antibody were not pre-mixed but added sequencially to the cell culture media. When supernatants of induced E. coli cultures were used in the assay, 65 μL of cell culture media containing 10 % serum and fluorescently labeled NTA-liposomes were mixed with 35 μL of supernatants. To test the internalization of monoclonal antibodies, which do not contain a (His)6-tag, 10 μg/mL of Protein A-(His)6 was used to complex 40 μg/mL of anti-ErbB2 mAB Herceptin. To strip cell surface of non-internalized liposome/ligand complexes, cells were washed 3–4 times with 170 μL PBS containing 2 mM MgCl2, 2 mM CaCl2, and 1 mM EDTA or with 250 mM phosphate buffered imidazole (pH 7.4), causing dissociation of the (His)6-Ni-NTA bond in the surface-bound liposome-ligand complexes. Cells were then lysed in 0.01 M NaOH (50 μL) before the fluorescence was read in a FL600 microfluorimeter (BIOTEK) using bandpass filters at 460/35 nm for excitation and 530/20 nm for emission. To quantify the amount of liposomes internalized by the cells, the aliquots of HPTS-loaded Ni-NTA liposomes containing known amounts of liposome phospholipid were lysed, and the standard curve of the marker fluorescence vs. liposome concentration was obtained in a similar manner. To study co-localization with transferrin, Ni-NTA-liposomes, anti-ErbB2-scFv-F5-(His)6 (20 μg/mL), and transferrin-phycoerythrin (10 μg/mL; obtained from Molecular Probes) were co-incubated for two hours with SKBR3 cells before observing cellular localization by fluorescence microscopy using a dual-pass filter.

### Quantification of surface bound anti-ErbB2-scFv-F5-(His)6 by flow cytometry

Cells were harvested by trypsinization using standard techniques. Anti-ErbB2-scFv-F5-(His)6 was incubated in triplicate with 1 × 10^5 ^cells in 96-well plates with V shaped wells for two hours at concentrations indicated. Cell staining with anti-ErbB2-scFv-F5-(His)6 was performed as described elsewhere [54] and fluorescence was measured by flow cytometry in using a FACSort cytofluorometer (Becton-Dickinson) and median fluorescence (F) was calculated using Cellquest software (Becton-Dickinson) and the background fluorescence with cells only were subtracted.

### Synthesis of 6-(1,2-distearoyl-sn-glycerophosphoryl-ethanolaminocarbonyl)-poly(oxyethylene)-oxycarbonyl)amino-2-(N, N-bis-carboxymethylamino)hexanoic acid nickel salt (DSPE-PEG-NTA-Ni)

6-Amino-2-(N, N-bis-carboxymethylamino)-hexanoic acid (N, N-biscarboxymethyl-L-lysine) was prepared as previously described [44], except that the removal of CBZ prospective group was in 4 M HBr/glacial acetic acid overnight, resulting in the recovery of the product as a hydrobromide. Distearoylphosphatidylethanolaminocarbonyl-poly(ethylene glycol)-propionic acid Nhydroxylsuccinimidyl ester (NHS-PEG-DSPE; 198 mg, 0.0445 mmol) prepared from poly(ethylene glycol) (MW 3,400, Shearwater Polymers, Huntsville, AL) was dissolved in the mixture of anhydrous ethanol (1 ml) and anhydrous chloroform (0.5 ml), mixed with a solution of 6-amino-2-(N, N-bis-carboxymethylamino)hexanoic acid hydrobromide (40.8 mg, 0.120 mmol) in 0.5 ml of anhydrous ethanol and 0.15 ml of triethylamine (1.08 mmol), and stirred for 2 h at 60°C. The reaction mixture was clarified by centrifugation at 15,500 × g for 5 min and clear supernatant was vacuum dried.and dissolved in 3 ml of 0.14 M NaCl. The mixture was clarified by centrifugation at 15,500 × g for 5 min, and the clear supernatant was vacuum dried. The residue was dissolved in 2.5 ml of 0.144 M NaCl, pH was adjusted to 6.8 with 1 M NaOH, and 0.12 ml of 1 M NiSO4 was added. The solution was applied to a 13 ml chromatography column with cross-linked dextran beads (Sephadex G-75, (Pharmacia Amersham, USA) using 0.144 M NaCl as eluent. The fractions appearing at the void volume (total 4 ml) were collected, and lyophilized overnight. The lyophilized cake was extracted with a mixture of 2 ml anhydrous ethanol and 0.2 ml chloroform; the insoluble matter was removed by centrifugation, and the clear solution was vacuum dried. The residue was re-dissolved in 2 ml of ethanol containing 0.1 ml chloroform, the solution clarified by centrifugation (15,500 × g, 5 min), and vacuum dried. Yield was 92 mg which represents 46 % of the theoretical yield. The product representing a bluish solid was soluble in a chloroform-methanol mixture (60:40, vol:vol) and in water, giving light blue solutions. The intended structure (fig. 4) was confirmed by 1H-NMR.

### Cytotoxicity studies

Specific cytotoxicity of ErbB2-targeted immunoliposomes containing various anti-cancer drugs was evaluated in SKBR3 cells plated at a density of 5,000 cells per well in 96-well plates and allowed to grow overnight. Immunoliposomes or control treatments were applied for 8 h at 37°C, followed by washing with PBS and re-adding growth media. Cells were incubated for an additional three days at 37°C and analyzed for cell viability using a calcein-AM cytotoxicity assay [55,56]. Briefly, following the three-day incubation, the media was removed and the cells washed once with 200 μl of phosphate buffered saline (PBS). Subsequently, PBS (100 μl) was added to each well and mixed gently with 100 μl of a freshly prepared calcein-AM stock (1 μM) in PBS. The cells were then allowed to incubate for 45 min at 37°C in a CO2 incubator. Following the incubation period, the cells were washed once with PBS (100 μl). A final volume (200 μl) of PBS was added to the cells and the amount of calcein produced by the cells was analyzed by measuring the fluorescence in a BioTek (FL600) fluorescence microtiter plate reader using 485/35 and 530/20 nm bandpass filters for excitation and emission, respectively. The amount of calcein fluorescence was normalized to that of non-treated cells and expressed as % cell viability.

## Abbreviations

CLIA Chelated Ligand Internalization Assay

DSPC distearoylphosphatidylcholine

HPTS 8-hydroxypyrene-1,3,6-trisulfonic acid (pyranine)

DOGS-NTA-Ni 1,2-dioleoyl-sn-glycero-3-[N(5-amino-1-carboxypentyl) iminodiacetic aicd]succinyl] (nickel salt)

DSPE-PEG-NTA-Ni 6-(1,2-distearoyl-sn-glycerophosphoryl-ethanolaminocarbonyl)-

poly(oxyethylene)-oxycarbonyl)amino-2-(N, N-biscarboxymethylamino)

hexanoic acid (nickel salt)

NTA nitriloacetic acid

PBS phosphate buffered saline

PEG-DSPE N- [methoxy(polyethylene glycol)-2000]-1,2-distearoyl-

Phosphatidylethanolamine

POPC 1-palmitoyl-2-oleoyl-phosphatidylcholine

## Authors' contributions

UN designed and performed the majority of the experiments described in the manuscript.

DK designed the liposomes that form the basis of the assay. DD synthesized some of the lipids described, constructed the drug containing liposomes, and performed the cytotoxicity assays. EP expressed the antibodies used in the manuscript. JM coordinated the project and outlined the experimental systems. All authors read and approved the final manuscript.
